# Posttraumatic stress and depressive symptoms in children after the Wenchuan earthquake

**DOI:** 10.1080/20008198.2018.1472992

**Published:** 2018-05-18

**Authors:** Jin Cheng, YiMing Liang, Lin Fu, ZhengKui Liu

**Affiliations:** a CAS Key Laboratory of Mental Health, Institute of Psychology, Beijing, China; b Department of Psychology, University of Chinese Academy of Sciences, Beijing, China

**Keywords:** PTSD, depressive symptoms, cross-lagged analysis, longitudinal study, natural disaster, TEPT, síntomas depresivos, análisis cruzado, estudio longitudinal, desastres naturales, PTSD, 抑郁症状, 交叉滞后分析, 纵向研究, 自然灾害, • The results showed a changed relationship between PTSD and depressive symptoms., • Our study suggests that females with a high degree of trauma exposure and poor parental relationship are the most important group that requires intervention., • The object of children’s posttraumatic therapy could be the entire family rather than only the children., • Future studies could collect larger samples and could distinguish between females and males and between different ages of children.

## Abstract

**Background:** Many studies have reported the comorbidity of posttraumatic stress disorder (PTSD) and depression in children. However, the underlying relationship between PTSD and depression remains unclear.

**Objective:** This study examines the relationship between PTSD and depressive symptoms in children who survived the Wenchuan earthquake in China.

**Methods:** In total, 301 children were assessed at four months and then followed up at 29, 40 and 52 months after the disaster. The ages of the children ranged from 9.6–14.6 years old, and the sample included 157 males and 144 females. The children were assessed by using the University of California at Los Angeles PTSD reaction index for DSM-IV for PTSD symptoms and the Children’s Depression Inventory for depressive symptoms.

**Results:** Comorbid PTSD and depressive symptoms were prevalent in 4.0, 3.3, 3.7 and 5.1% of the participants at times 1, 2, 3 and 4, respectively. The cross-lagged analysis indicated that PTSD symptoms at time 1 predicted depressive symptoms at time 2; depressive symptoms at time 1 predicted PTSD symptoms at time 2; depressive symptoms at time 2 predicted PTSD symptoms at time 3; and depressive symptoms at time 3 predicted PTSD symptoms at time 4. The findings also showed that being female, poor parental relationships and trauma exposure were risk factors for PTSD or depressive symptoms.

**Conclusions:** The results suggest that the causal relationship between PTSD and depressive symptoms changes over time; the effects of PTSD symptoms tend to decrease, while those of depressive symptoms tend to increase. Two stages of the relationship between PTSD and depressive symptoms were observed, namely, that PTSD and depressive symptoms first influenced each other and then that depressive symptoms predicted PTSD. The results of our study also suggest that females with poor parental relationships and a high degree of trauma exposure are more likely to require intervention.

## Abstract (es)

**Antecedentes:** Muchos estudios han reportado la comorbilidad del trastorno de estrés postraumático (TEPT) y depresión en niños. Sin embargo, la relación subyacente entre TEPT y depresión permanece poco clara.

**Objetivo:** Este estudio examina la relación entre TEPT y síntomas depresivos en niños que sobrevivieron al terremoto de Wenchuan en China.

**Métodos:** En total, 301 niños fueron evaluados a los 4 meses y luego en seguimiento a los 29, 40 y 52 meses después del desastre. Los rangos de edades de los niños fueron entre 9,6-14,6 años y la muestra incluyó 157 hombres y 144 mujeres. Los niños fueron evaluados usando el índice de reacción de TEPT de la Universidad de California en Los Ángeles para los síntomas de TEPT según el DSM-IV y para los síntomas depresivos el Inventario de Depresión para Niños.

**Resultados:** La comorbilidad TEPT y síntomas depresivos tuvieron una prevalencia de un 4,0%, 3,3%, 3,7% y 5,1% de los participantes a los tiempos 1, 2, 3 y 4, respectivamente. El análisis cruzado indicó que los síntomas de TEPT en el tiempo 1 predijo los síntomas depresivos al tiempo 2, y que los síntomas depresivos al tiempo 1 predijeron síntomas de TEPT al tiempo 2; los síntomas depresivos al tiempo 2 predijeron síntomas de TEPT al tiempo 3; y síntomas depresivos al tiempo 3 predijeron síntomas de TEPT al tiempo 4. Los hallazgos también mostraron que ser mujer, malas relaciones parental y exposición a trauma fueron factores de riesgo para TEPT o síntomas depresivos.

**Conclusiones:** Los resultados sugieren que la relación causal entre TEPT y síntomas depresivos cambia en el tiempo; los efectos de los síntomas de TEPT tienden a disminuir, mientras que los síntomas depresivos tienden a aumentar. Se observaron dos estadíos de relación entre los síntomas de TEPT y depresivos, esto es, que primero el TEPT y síntomas depresivos se influyen mutuamente y luego, los síntomas depresivos predijeron el TEPT. Los resultados de nuestro estudio también sugieren que las niñas con malas relaciones parentales y alto grado de exposición a trauma son las que probablemente requieren mayor intervención.

## Abstract (zh)

**背景：**许多研究报告了儿童创伤后应激障碍（PTSD）和抑郁的共病。然而，PTSD和抑郁症之间的潜在关系仍不清楚。

**目的：**本研究探讨中国汶川地震幸存儿童中PTSD与抑郁症状之间的关系。

**方法：**对汶川地震幸存的301名儿童进行灾后4个月的随访，并在灾后29个月、40个月和52个月进行随访。 儿童的年龄在9.6到14.6岁之间，样本包括157名男性和144名女性。 通过使用加州大学洛杉矶分校的DSM-IV PTSD反应指数来评估儿童的PTSD症状，使用《儿童抑郁症量表》评估抑郁症状。

**结果：**在第1、2、3和4次随访期，共病PTSD和抑郁症状的患病率分别为4.0％，3.3％，3.7％和5.1％。交叉滞后分析表明，第1次随访的PTSD症状预测在第2次随访的抑郁症状，第1次随访时的抑郁症状预测第2次随访的PTSD症状; 第2次随访的抑郁症状预测第3次随访的PTSD症状; 第3次随访的抑郁症状预测了第4次随访的PTSD症状。研究结果还表明，女性、较差的父母关系和创伤暴露是PTSD或抑郁症状的危险因素。

**结论：**结果表明PTSD与抑郁症状之间的因果关系随时间而变化; PTSD症状的影响趋于减少，而抑郁症状的影响趋于增加。 观察到PTSD和抑郁症状之间存在两个阶段的关系，即PTSD和抑郁症状首先相互影响，然后抑郁症状预测PTSD。 我们的研究结果还表明，与父母关系不佳和高度创伤暴露的女性更可能需要干预。

## 1. Introduction

The symptoms of posttraumatic stress disorder (PTSD) often co-occur with other psychiatric disorders, such as depression, generalized anxiety disorder, social phobia, simple phobia and panic disorder (Kessler, Sonnega, Bromet, Hughes, & Nelson, 1995; Krueger & Markon, 2006). Empirical findings have demonstrated a high rate of comorbidity between PTSD and depressive symptoms (O’Donnell, Creamer, & Pattison, 2004; Tekin et al., 2016). Ramsawh et al. (2014) found that individuals with both PTSD and depression were 2.6 times more likely to have past-year suicidality than those with either diagnosis alone.

The underlying relationship between the co-occurrence of PTSD and depressive symptoms remains unclear. The potential mechanisms of this relationship include three models: the depressogenic model, the demoralization model and the synchronous change model (Schindel-Allon, Aderka, Shahar, Stein, & Gilboa-Schechtman, 2010). The depressogenic model suggests that depressive symptoms may predict subsequent PTSD symptoms (Breslau, Davis, Andreski, & Peterson, 1991; Merriman, Norman, & Barton, 2007). The demoralization model suggests that PTSD symptoms are the causes of depressive symptoms (Mangelli et al., 2005). The synchronous change model suggests that PTSD and depression may be influenced by a third variable and show no real association with each other (Breslau & Schultz, 2013; Vinck, Pham, Stover, & Weinstein, 2007; Wright et al., 2011). In a twin study of 6609 members of the Vietnam Era Twin Registry (male–male twins), no residual genetic and environmental variance overlapped between combat exposure and major depression after controlling for variance common to PTSD (Scherrer et al., 2008). These findings suggest that PTSD may play a mediating role in depression following trauma. Nonetheless, both determining the mechanism behind this comorbidity and identifying which condition is the cause and which is the result are difficult.

Many studies have reported the comorbidity of PTSD and depression in children (Adams et al., 2015; Kar & Bastia, 2006; Thabet, Abed, & Vostanis, 2004). In one cross-sectional study, 68.4% of adolescents with major depression suffered PTSD 14 months after a cyclone (Kar & Bastia, 2006). A total of 2000 adolescents (aged 12–17 years), 4–13.5 months after a tornado, were interviewed and the comorbidity of PTSD and depression was 3.7% (Adams et al., 2015). During a war conflict, 23.9% of 403 children (aged 9–15 years) suffered from both PTSD and depression (Thabet et al., 2004). Some longitudinal studies have also examined the relationship between PTSD and depression among children and adolescents. A 5–8-year longitudinal study on the onset of PTSD and depression was conducted among 216 adolescents after a shipping disaster (Bolton, O’Ryan, Udwin, Boyle, & Yule, 2000); no causal relationship was determined. Another five-year longitudinal investigation of PTSD treatment among adolescents revealed that it also relieved depressive symptoms (Goenjian et al., 2005), but the study lacked a group with treatment for depressive symptoms. However, the results of two other studies using cross-lagged analyses among children after earthquakes demonstrated conflicting results (Fu, Cheng, & Liu, 2017; Ying, Wu, & Lin, 2012). To date, there is no consistent conclusion about the relationship between PTSD and depression in children or adolescents.

An interactive model has been proposed to describe the course of PTSD and depression after traumatic events (Goenjian et al., 1995). The model is based on both children and adolescents who survived an earthquake. This model suggests that exposure to trauma directly causes PTSD among affected individuals and that PTSD predicts depressive symptoms after exposure to the event. Additional important aspects of the interactive model include depressive symptoms influencing PTSD and depressive symptoms and PTSD symptoms subsequently influencing each other. Two longitudinal studies have shown that PTSD and depressive symptoms predict each other in the cross-lagged model and give support, in part, to the interactive model (Erickson, Wolfe, King, King, & Sharkansky, 2001; Horesh, Lowe, Galea, Uddin, & Koenen, 2015). According to the view of the interactive model, the three other models mentioned above may not completely conflict with one another. Each of these models is likely to exist in different periods during the developmental course of PTSD and depression. Furthermore, many researchers are interested in changes in the relationship between PTSD and depressive symptoms over time, but no empirical study has revealed changed relationships after all types of traumatic incidents.

This interactive model provides a new perspective on the relationship between PTSD and depression by suggesting that this relationship changes over time. Thus, time is most likely a key element in the model, possibly for the following reasons. First, the relationship between PTSD and depression in the model is considered to develop after traumatic events. Because chronic stress occurs without a precise beginning or end, the relationship between PTSD and depression can be assessed in the context of an independent and potentially traumatic event. Second, hypothesis testing in the model requires a longitudinal design, which necessitates an early start and a long follow-up period. Longitudinal investigations are often limited in this aspect; as a result, studies have evaluated parts of the model rather than the model as a whole. These issues can be resolved only by longitudinal studies that start early, track individuals over a long period and conduct multiple assessments.

In addition to time, different trauma types and participants may also influence the relationship between PTSD and depression. Potentially traumatic events include manmade and natural disasters. Compared to manmade disasters (i.e. combat or physical violence), natural disasters (i.e. earthquakes or floods) lead to fewer PTSD symptoms among survivors (Bromet et al., 2017; Kessler et al., 2017). In addition, there are psychological differences between children and adults. Childhood and adolescence are unique periods of growth and development that are the foundations of adulthood. Children and adolescents are easily exposed to second-hand trauma (TV and nonprofessional psychological first aid), which has been shown to increase the risk of PTSD among adolescents (Yeung et al., 2016). In contrast, a systematic review of PTSD reactions after the Wenchuan earthquake showed that children and adolescents had a lower prevalence of PTSD and that their symptoms lasted for a much shorter period than did those of adults (Hong & Efferth, 2016). These findings could influence the different relationships between PTSD and depression in children and adults. Many other factors are related to PTSD and depression, including trauma exposure, age and gender (Kilpatrick et al., 2003; Thabet et al., 2004; Thienkrua et al., 2006). In addition to those variables, some investigations have shown that the parental relationship can affect PTSD or depressive symptoms in children (Bokszczanin, 2008; Dekel & Monson, 2010; Lauterbach et al., 2007). Thus, these variables should be considered when exploring the relationship between PTSD and depression.

The present study aims to bridge the knowledge gap regarding the co-occurrence of PTSD and depressive symptoms using a longitudinal design. The participants are survivors of the Wenchuan earthquake, considered one of the deadliest and most devastating natural disasters to have ever occurred in China. This study was specifically designed to examine the relationship between PTSD and depressive symptoms using cross-lagged, latent variable, structural equation modelling (SEM) and to test the four abovementioned models in children who survived the earthquake. Based on the available evidence, we expected to find support for the interactive model. As stated above, although common factors may influence depression and PTSD, PTSD may still affect depression, or depression may have a further effect on PTSD. Furthermore, their relationship is likely to change over time.

## 2. Materials and methods

### 2.1. Participants

**Figure 1.**
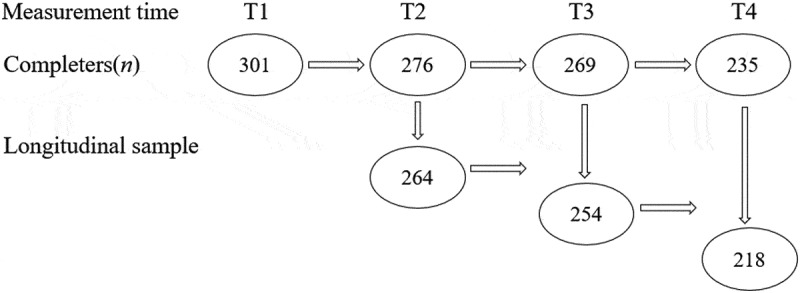
Details of the longitudinal sample.

A magnitude 8.0 earthquake occurred in Wenchuan, China, on 12 May 2008. By 25 September 2008, the total death toll for this disaster had reached 69,227. Additionally, 374,643 individuals were injured and 17,923 were recorded as missing (Zhu, 2008). Between 11 September and 16 September 2008, 326 fourth- and sixth-grade children were recruited using convenience sampling from the Zhongguo Kexueyuan Qingnian Beichuan Xiwang school located in Wenchuan County, which was the most severely affected area. These school buildings were newly built by the Chinese Academy of Sciences and did not collapse during the earthquake (Li, 2008). In total, one student died and 12 students were injured. A set of checklists capturing PTSD and depressive symptoms were administered to all participants. Overall, 301 children (92%) completed the checklists at baseline between 11 September and 16 September 2008 (approximately four months after the earthquake, time 1, T1). The children were followed up from 13 October to 4 November 2010 (approximately 29 months after the earthquake, time 2, T2), from 10 September to 30 September 2011 (approximately 40 months after the earthquake, time 3, T3), and from 18 September to 3 October 2012 (approximately 52 months after the earthquake, time 4, T4). In total, 276 (84%) children completed the second assessment, 269 (83%) completed the third assessment and 235 (72%) completed the fourth assessment. Figure 1 shows the detailed numbers of the longitudinal participants at the four time points. The participants (*n *= 301) ranged in age from 9.6 to 14.6 years (*M *= 10.9, *SD *= 1.08) at T1.

Because some students transferred to other schools, entered high school or dropped out of school, they could not be contacted or assessed. The results from χ^2^ and *t*-tests (two-tailed) showed that the drop-outs at the following three time points did not statistically significant differ from the completers by gender (T2, χ^2^ = 3.71, *p* = .054; T3, χ^2^ = 2.0, *p* = .15; T4, χ^2^ = .35, *p* = .55), age (T2, χ^2^ = 5.07, *p* = .41; T3, χ^2^ = 5.93, *p* = .41; T4, χ^2^ = 8.1, *p* = .15), parental relationship (T2, χ^2^ = 2.94, *p* = .40; T3, χ^2^ = 4.36, *p* = .22; T4, χ^2^ = 6.05, *p* = .11), trauma exposure (T2, χ^2^ = 5.57, *p* = .35; T3, χ^2^ = 5.84, *p* = .32; T4, χ^2^ = .61, *p* = .99), T1 PTSD scores (T2, *t* = 1.04, *p* = .30; T3, *t *= .50, *p* = .62; T4, *t* = .31, *p* = .76) or T1 depressive symptoms (T2, *t* = 1.63, *p* = .10; T3, *t *= .49, *p* = .63; T4, *t* = 1.73, *p* = .08). Further, we conducted analyses using Little’s Missing Completely at Random (MCAR) test. The result showed that the missing data were not completely at random (χ^2^ = 24,453, *df* = 24,051, *p* = .034).

In the survey, the participants were assessed collectively using questionnaires in a group format at the end of class at T1; their teachers were present in the classrooms at the time of the assessment. The interviewers included a postgraduate and a volunteer, both of whom were uniformly trained using the same standardized instructions and tips. Because the children transferred to another school or dropped out of school, data were collected at T2, T3 and T4 using questionnaires at the children’s homes in the presence of their guardians by the interviewers (a postgraduate and a volunteer) within two weeks. The parents were considered the children’s guardians at home, while the children’s teachers and headmasters were considered their guardians in school. Written informed consent was obtained from the participants and their parents/teachers (at home/school) after clearly informing the participants that they were not required to respond to any of the questions. The parents were informed of the longitudinal study design, and verbal agreement was obtained from the parents at each time point. Students could ask questions if they did not understand the survey. The study design and procedures were approved by the ethics review committee of the Institute of Psychology of the Chinese Academy of Sciences.

### 2.2. Measures

The University of California at Los Angeles (UCLA) PTSD reaction index for DSM-IV, version 1 (UCLA PTSD-RI; Steinberg & Brymer, 2008; Steinberg, Brymer, Decker, & Pynoos, 2004) was administered to all participants at all time points. The reliability and validity of the Chinese version of the UCLA PTSD-RI have been demonstrated (Fu, Cheng, Wu, & Liu, 2018). The internal consistency of the UCLA PTSD-RI was measured; Cronbach’s *α *= .90, and Cronbach’s *α* for the subscales ranged from .74 to .80 in Fu et al.’s study (2018). The UCLA PTSD-RI is a 20-item scale that assesses PTSD symptoms in children regarding three factors: re-experiencing, avoidance and hyperarousal. The items are scored on a 5-point scale ranging from 0 (not at all) to 4 (very much), and the children select the statement that best characterizes them during the past month. A participant was considered to have PTSD if he/she had a total UCLA PTSD-RI score greater than or equal to 38. The internal consistency (Cronbach’s α) of the UCLA PTSD-RI in this study was .86 at T1, .88 at T2, .92 at T3 and .91 at T4.

The Children’s Depression Inventory (CDI; Kovacs, 1992; Wu, Lu, Tan, & Yao, 2010) is a 27-item scale that assesses depression in children and consists of five subscales: anhedonia, negative mood, negative self-esteem, ineffectiveness and interpersonal problems. On a 3-point scale ranging from 0 to 2, participants are asked to choose the statement that best characterizes them during the preceding two weeks. Wu et al. (2010) tested the reliability and validity of the CDI among Chinese children (Wu et al., 2010). The internal consistency of the CDI was measured in Wu et al.’s study (2010); Cronbach’s *α* was .88 for the total CDI and ranged from .60 to .74 for the subscales. Timbremont, Braet, and Dreessen (2004) suggested that a cut-off score of 19 was adequate for the general screening of depression (Timbremont et al., 2004). The internal consistency (Cronbach’s α) of the CDI in this study was .88 at T1, .89 at T2, .91 at T3 and .91 at T4.

Additionally, items to capture demographics, trauma exposure and parental relationships were included as control variables. Five trauma exposure questions were assessed using true/false (scoring 1/0) response options; these questions assessed whether the respondents had ‘been trapped’, ‘been injured’, ‘witnessed buildings falling’, ‘lost a relative’ or ‘witnessed or touched a dead body’ during the earthquake. A general score for trauma exposure was computed by summing these item scores. Regarding their parental relationships, the participants were asked one question: ‘How is your parental relationship?’ Four response options were provided: ‘very good’, ‘good’, ‘average’ and ‘bad’ (scoring 1–4).

### 2.3. Data analyses

First, a measurement model based on the sum scores was established using confirmatory factor analysis (CFA). PTSD and depressive symptoms were combined in the CFA. The model included eight latent factors (depression and PTSD symptoms at T1, T2, T3 and T4). Then, the longitudinal measurement invariance analysis within the framework of the CFA was conducted with the MPLUS 7.0 program. Configural invariance and metric invariance were checked. If configural invariance (baseline model) was supported, further restrictive constraints could be imposed on the model because the model was conducted in the conventional multiple group CFA invariance test. Factor loadings were constrained to be equal across time to test metric invariance. In addition, correlations were estimated among all possible pairs of residual error variance among T1, T2, T3 and T4. Finally, SEM was used to analyse the relationship between depression and PTSD by testing the four models. All cross-lagged effects were constrained to zero to test the synchronous change model. This model is a residual change model analysing relative change, and absolute change is not in focus in this analysis. Only the effects of PTSD symptoms on depressive symptoms were used to test the demoralization model, and only the paths from depressive symptoms to PTSD symptoms were used to test the depressogenic model. Both the effects of PTSD symptoms on depressive symptoms and the effects of depressive symptoms on PTSD symptoms were used to test the interactive model. The depressogenic and demoralization models included synchronous and lagged paths. Moreover, to account for the complexity of the models, correlations were specified between the residuals in these latent factors; autocorrelations were not allowed between the error terms of the manifest factors across or within waves. The Bayesian information criterion (BIC) and other fit measures were considered when comparing the models. The best-fitting model was selected.

Demographic characteristics (e.g. age and gender), trauma exposure and parental relationships were included as predictors of PTSD and depression scores at T1. All analyses were conducted in MPLUS 7.0 using the robust maximum likelihood (MLR) iteration procedure. The model fit in this study was assessed using the following fit indices: χ^2^/*df* index, Tucker-Lewis Index (TLI; Bentler, 1990), comparative fit index (CFI; Bentler, 1990), root mean square error of approximation (RMSEA; Hu & Bentler, 1998) and the standardized root mean square residual (SRMR; Hu & Bentler, 1998). Cut-offs for an acceptable model fit were set at < 3 for χ^2^/*df, <* .08 for the RMSEA and *>* .90 for the CFI and TLI (Bentler, 1990; Hu & Bentler, 1998, 1999; Kline, 2015). The corrected scaled Chi-square difference test and changes in the CFI were used to compare nested models. A Chi-square difference test was conducted to assess whether the baseline model was significantly different from the constrained model. A nonsignificant Chi-square difference test indicated that factor loadings were invariant across time, which satisfied metric invariance. A CFI difference value < .01 indicated that the measurement invariance hypothesis should not be rejected; mean differences exist when CFI differences range from .01 to .02, and definite differences exist when CFI differences are > .02 (Cheung & Rensvold, 2002).

All data for the 301 students were used in subsequent analyses. With the exception of five participants who did not report any trauma exposure, no missing data were found for the predictors. The five missing data points were deleted from the analyses. Missing data were addressed using full information maximum likelihood (FIML) estimates in MPLUS 7.0. The results produced using the FIML method were superior to those obtained using conventional methods for handling missing data, because FIML was subject to less bias and was more reliable (Newman, 2003). Coefficients were standardized or unstandardized throughout the manuscript. In this study, *p *< .05 means statistical significance. All of the results are standardized regression weights.

## 3. Results

### 3.1. Descriptive results

The demographic characteristics and other control variables of the sample are shown in Table 1. According to the criterion, cut-off scores of 38 and 19 were used for probable PTSD and depression, respectively. The prevalence of probable PTSD at T1, T2, T3 and T4 was 9.3, 4.3, 4.1 and 5.5%, respectively. The prevalence of probable depression at T1, T2, T3 and T4 was 34.2, 26.5, 30.5 and 26.0%, respectively. Finally, comorbid probable PTSD and depression at T1, T2, T3 and T4 was prevalent in 4.0, 3.3, 3.7 and 5.1% of the participants, respectively.

**Table 1.** Descriptive statistics (*n* = 301).

	*N*	%	Mean (*SD*)	Min	Max
Total	301	100			
Gender					
Male	157	52.2			
Female	144	47.8			
Age (years)	295	98	10.9 (1.1)	9.6	14.6
Grade					
Fourth	116	38.5			
Sixth	185	61.5			
Parental relationship					
Very good	207	68.8			
Good	49	16.3			
General	33	11.0			
Bad	12	4.0			
Trauma exposure	296	96.7	2.5 (1.2)	0	5
Witnessing a dead body	159	52.8			
Witness a building collapse	218	72.4			
Lost one (or more) relative(s)	244	81.1			
Was trapped	33	11.0			
Was injured	99	32.9			

The scores and correlations of PTSD and depressive symptoms at the three time points are available in Supplemental data Table S1. All of the correlations were significant and ranged from .30 to .80 (*p *< .001). The correlations between PTSD and depressive symptoms at T1, T2, T3 and T4 were .51, .70, .67 and .74, respectively. The correlations between the PTSD symptoms at all four time points ranged from .41 to .68, and those of the depressive symptoms ranged from .38 to .78. The correlations between the CDI scores and the three subscales of the UCLA PTSD-RI (re-experiencing, avoidance and hyperarousal) ranged from .23–.50, .26–.62 and .21–.57, respectively. No significant statistical differences were observed in the correlations among the CDI scores and three subscales.

### 3.2. Measurement model and measurement invariance

The measurement model showed a good fit (χ^2^ = 832.975, *df* = 436, χ^2^/*df* = 1.9, TLI = .891, CFI = .910, RMSEA = .055). The loadings of the manifest variables on their corresponding latent factors were all significant and ranged from .47 to .90 (Supplemental data Table S2).

The test of measurement invariance showed that both the configural invariance and the metric invariance of PTSD and depression were supported. The parameters of the model are shown in Supplemental data Table S3.

### 3.3. Structural model results

The parameters of the four models are shown in Table 2. All fit measures showed that the interactive model provided the best fit. The depressogenic model did not support the notion that depressive symptoms predicted PTSD symptoms; the demoralization model also did not support the notion that PTSD symptoms predicted depressive symptoms (Supplemental data Figures S1 and S2).

**Table 2.** Parameters of the four models.

	RMSEA	SRMR	CFI	TLI	BIC	Adjusted BIC	χ^2^	*df*
Synchronous change model	.059	.137	.871	.858	34621.86	34241.31	1149.46	568
Depressogenic model	.055	.068	.886	.875	34539.37	34168.32	5118.32	624
Demoralization model	.055	.070	.884	.874	34546.43	34175.39	5118.32	624
Interactive model	.055	.062	.888	.877	34545.29	34164.73	5118.32	624

**Figure 2.**
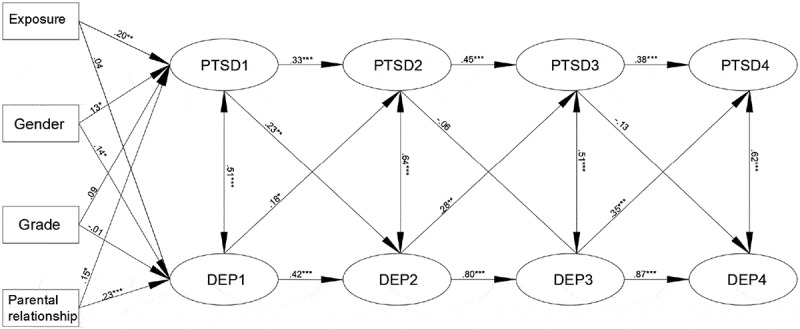
The cross-lagged SEM of PTSD and depression. Notes: PTSD = posttraumatic stress disorder; DEP = depression; 1 = the first assessment four months after the earthquake; 2 = the second assessment 2.5 years after the earthquake; 3 = the third assessment 3.5 years after the earthquake; 4 = the fourth assessment 4.5 years after the earthquake.* *p *< .05** *p *< .01*** *p *< .001

The interactive model was adjusted to optimize the fit (RMSEA = .048, SRMR = .062, CFI = .92, TLI = .91, χ^2^ = 5118.32, *df *= 624, see Figure 2) and ensured that the adjustment did not change the paths. The following significant paths between the latent factors were found.

Regarding the stability effects, the stability of PTSD symptoms tended to decrease across time, while that of the depressive symptoms tended to increase. Four significant paths were observed for the cross-lagged effects: T1 PTSD symptoms predicted T2 depressive symptoms (β = .23, 95% confidence interval (CI) = [.03, .43], *p* < .01), and T1 depressive symptoms predicted T2 PTSD symptoms (β = .18, 95% CI = [-.04, .40], *p* < .05); T2 depressive symptoms predicted T3 PTSD symptoms (β = .28, 95% CI = [.02, .53], *p* < .01); and T3 depressive symptoms predicted T4 PTSD symptoms (β = .35, 95% CI = [.12, .58], *p* < .001).

The impact of gender, grade, parental relationship and degree of trauma exposure were considered in the model. The results revealed that the degree of trauma exposure significantly predicted PTSD symptoms (β = .20, 95% CI = [.04, .37], *p* < .01) but not depressive symptoms, females had more PTSD and depressive symptoms than did males (β = .13, 95% CI = [-.03, .29], *p *< .05; β = .14, 95% CI = [-.02, .30], *p *< .05, respectively, male = 1, female = 2) and children with poor parental relationships had more PTSD and depressive symptoms than did children with good parental relationships (β = .15, 95% CI = [-.01, .31], *p* < .05; β = .23, 95% CI = [.08, .39], *p *< .05).

## 4. Discussion

The interactive model describing the comorbidity of PTSD and depressive symptoms resulted in the best-fit model. The results showed a changed relationship between PTSD and depressive symptoms over time. The model showed a mutual effect between PTSD and depressive symptoms during the early period, whereas the depressogenic model was supported during the later period. However, our findings did not show that PTSD symptoms influenced depressive symptoms more than 2.5 years post-earthquake. Thus, our results partially support Goenjian’s interactive model. The change in the relationship between PTSD and depressive symptoms may result in the stability and course of PTSD symptoms. Our results showed that the stability of depressive symptoms tended to increase over time, while the stability of PTSD symptoms tended to decrease. The probability of PTSD decreased over time after the earthquake (9.3–5.5%). In summary, the changed relationship in our study included two stages, namely, the PTSD and depressive symptoms influenced each other and then the depressive symptoms predicted PTSD symptoms.

However, the relationship between PTSD and depressive symptoms may have been different in the early days (before four months) or more than 4.5 years after the disaster. For example, our findings were not consistent with those of two related studies of Chinese children who survived the earthquake (Fu et al., 2017; Ying et al., 2012). In Fu et al.’s study (2017), the authors followed 197 children (aged 10–14 years) at two weeks (assessing acute stress symptoms and depressive symptoms) and 1.5 months (assessing PTSD and depressive symptoms) after the Lushan earthquake; the results showed that acute stress symptoms predicted PTSD and depressive symptoms, while depressive symptoms at two weeks did not affect PTSD symptoms at 1.5 months. Our investigation and the study of Ying et al. (2012) were similar but were conducted independently of each other. In both studies, the participants were children, and the traumatic event was the Wenchuan earthquake, which occurred on 12 May 2008 (Ying et al., 2012). However, the measurement time points in the Ying et al. (2012) study occurred at 1, 1.5 and 2 years after the event. The major difference was that our study began at four months after the event, which was considerably earlier than the start date of their study (four months vs. one year). Ying et al. (2012) detected only the significant paths from depressive symptoms to PTSD. The difference in the measurement time likely accounts for the differences in the results of these three studies. The measurement time may be more important than previously considered. In conclusion, three stages may exist in the relationship between PTSD and depression as time passes after a traumatic event. In the first stage, PTSD occurs early, which predicts depression; in the second stage, PTSD and depression have an interactive effect; and in the third stage, depression prevents recovery from PTSD. Overall, the changed relationship between PTSD and depression indicated that the effect of PTSD symptoms tended to decrease, while the effect of depressive symptoms tended to increase. In the present study, the probability of PTSD after the Wenchuan earthquake was relatively lower than reports in other studies. Specifically, the probability of PTSD ranged from 15.9 to 36.5% among children in some studies conducted from three months to three years after the Wenchuan earthquake (Fan, Zhang, Yang, Mo, & Liu, 2011; Hou et al., 2011; Pan et al., 2015), which was higher than the probability obtained in our study, whereas the probability of PTSD one year later (5.6–8.7%) found in other studies was similar to our result (Lin et al., 2013; Tian, Wong, Li, & Jiang, 2014; Ying, Wu, & Chen, 2013). There were two main reasons for this finding. First, few deaths and injuries occurred in the Zhongguo Kexueyuan Qingnian Beichuan Xiwang school. The school was called the miracle school because it did not collapse during the Wenchuan earthquake despite being at the epicentre (Li, 2008). Second, all students accepted school-based, post-disaster psychological therapy. After the Wenchuan earthquake, mental health courses were conducted by the Institute of Psychology, Chinese Academy of Sciences, in this school and some other temporary shelters (Chinese Academy of Sciences, 2008) beginning on 15 May 2008 (Shi & Yan, 2011) and lasting for more than five years (Shi, Fu, & Yan, 2017). Although the probability of PTSD was relatively low in our study, PTSD symptoms still had an effect on depressive symptoms among children, with a relatively lower probability of PTSD. PTSD symptoms may have a stronger effect and depressive symptoms may have a weaker effect in the early stages of trauma than in the later stages. The reason may be that most of the participants’ PTSD symptoms did not reach the threshold, but the participants still suffered PTSD symptoms. PTSD symptoms among those participants would also contribute to predictions of their depressive symptoms and could be predicted by depressive symptoms.

The sequence of PTSD and depressive symptoms is important when considering the possible pathways. Based on the hypothesis of three stages, PTSD symptoms are the primary symptoms and depressive symptoms are the secondary symptoms after traumatic events. However, we did not collect data before the earthquake. An investigation that collected data from 137 undergraduates before the earthquake found that the depression scores of the undergraduate survivors 14 days before the earthquake did not differ significantly from their scores 10 days and seven weeks after the earthquake; however, their PTSD scores did differ significantly between 14 days prior to and 10 days after the event (Nolen-Hoeksema & Morrow, 1991). Furthermore, in terms of therapy, Meyer, Kimbrel, Tull, and Morissette (2011) found that treatments for PTSD tended to decrease symptoms of depression, whereas treatments for comorbid disorders such as depression did not necessarily decrease symptoms of PTSD (Meyer et al., 2011). Those findings support the hypothesis that PTSD precedes depression. An early measurement time point, a long analysis period and more measurement waves should be considered in longitudinal investigations to detect changes in this relationship.

Risk factors of PTSD and depressive symptoms were also observed in our study. First, the high degree of trauma exposure predicted high levels of PTSD symptoms but not depressive symptoms. The dose-effect relationship of trauma exposure to PTSD symptoms has been supported by many previous studies (Hong & Efferth, 2016). However, trauma exposure had no statistically significant effect on depressive symptoms in our study. The reason for this finding may be that PTSD acts as a mediator between trauma exposure and depressive symptoms (Koenen et al., 2003). If this reasoning is true, then PTSD symptoms are most likely the primary symptoms. Second, in our study, females had more severe PTSD and depressive symptoms than males did. These findings are consistent with the results of other studies reporting that females had more PTSD and depressive symptoms than did their male counterparts (Adams et al., 2015; Bokszczanin, 2007; Green et al., 1991). Furthermore, in our sample, children with poor parental relationships had a greater risk of PTSD and depressive symptoms. One systematic review suggested that the highest level of evidence linked greater interparental conflict with worse depression (Yap, Pilkington, Ryan, & Jorm, 2014). The existing studies revealed that poor family relations (i.e. family conflict or a poor parent–child relationship) predicted PTSD (Bokszczanin, 2008; Dekel & Monson, 2010). Finally, the grade of the children had no effect on PTSD or depressive symptoms, possibly because the two grades in this study were close (4th and 6th grades) and because the children were young (9.4–14.6 years old).

Nevertheless, this study has some limitations. First, our sample received psychological intervention after the earthquake. The Chinese government and NGOs offered interventions to survivors and people who needed help in the Sichuan area; in particular, there were many programmes for children and students. These programmes helped to remit PTSD and depressive symptoms among children (Goenjian et al., 2005), which likely influenced the relationship between PTSD and depressive symptoms. Second, no data were collected prior to the earthquake, which would have facilitated a pre- and post-event comparison of the mental health statuses, anxiety and general premorbidities. Therefore, whether PTSD causes the onset of depressive symptoms after traumatic events cannot be verified. Third, as suggested by Wolf, Harrington, Clark and Miller, our sample size was small for the cross-lagged SEM (Wolf, Harrington, Clark, & Miller, 2013). For convenience, we collected our sample from only one school. A larger sample should be included in future studies. Fourth, the measurement model and measurement invariance test were very important issues. Although both tests were well supported in the present study, the measurement model and measurement invariance were tested by the sum scores of the subscales and not by observed variables. Moreover, these issues were not discussed in detail due to space limitations. These important issues may be discussed specifically in a future study. Fifth, in this study, the missing data were not completely at random, although there was no difference between drop-outs and completers in terms of gender, age, trauma exposure, parental relationship and PTSD scores and depressive symptoms. These issues may still influence the accuracy of the results even though all available data were used, which ensures stronger generalizability of the results.

Despite these limitations, this study has significant theoretical and practical implications. The most significant finding was that this study observed a change in the relationship between PTSD and depression among children after the earthquake, which included two stages. In the first stage, PTSD and depressive symptoms influenced each other, whereas in the second stage, depressive symptoms predicted PTSD symptoms. However, one study with an early measurement time suggested that depressive symptoms did not influence PTSD, while acute stress symptoms predicted depressive symptoms (Fu et al., 2017). Another study with pretrauma data showed that depression was not significantly different between 14 days before and 10 days after the earthquake or between 14 days before and seven weeks after the earthquake among undergraduate survivors but that their PTSD scores did differ significantly between 14 days prior to and 10 days after the event (Nolen-Hoeksema & Morrow, 1991). Based on these studies, PTSD symptoms most likely preceded depressive symptoms, and PTSD symptoms most likely affected depressive symptoms in the earliest stage of their relationship. Future studies covering a longer period and including an early assessment point and more measurement waves are needed to determine the definite stages of the relationship between PTSD and depression and to attain a comprehensive understanding of the related mechanisms. These findings also supplement the empirical evidence of PTSD and depression comorbidity among children. Because children are in a critical developmental stage of life, further investigations among children are expected. Moreover, our study revealed a gender difference in PTSD and depressive symptoms. Other investigations also found that gender, age and trauma type affected PTSD and depressive symptoms (Ditlevsen & Elklit, 2012; Ehring & Quack, 2010). Future studies should use larger samples and distinguish between females and males and between different age groups of children. In addition, some studies have proposed a deeper investigation of the relationship between clusters of PTSD and depression (Horesh et al., 2017; Seligowski, Rogers, & Orcutt, 2016). In the present study, no significant statistical differences were found in the correlations among the three clusters and CDI scores; therefore, additional studies on this topic are needed. In conclusion, although our study provides a new perspective on the relationship between PTSD and depression, additional empirical studies are needed. The clinical implications of our findings provide some suggestions regarding the treatment sequence of symptoms and the specific groups. First, based on our findings of the stability of PTSD and depressive symptoms, treatment for PTSD might be a better choice in the early stage, whereas treatment for depression may be beneficial in the later period. Similar to many other studies, our study also suggests that females are more likely to require intervention post-disaster. In addition, because poor parental relationships predict children’s PTSD symptoms and the family is the child’s main environment, the object of a child’s posttraumatic therapy can be the entire family rather than only the child. One study reviewed the association between family relationships and PTSD and proposed a family-focused intervention (Bokszczanin, 2008). However, additional empirical studies are needed to test this hypothesis in the future. It is with hope that further progress will be made in the near future.
