# Supplementary material for: Posttraumatic stress and depressive symptoms in children after the Wenchuan earthquake
Source: Eur J Psychotraumatol. 2018 May 18;9(1):1472992. doi: 10.1080/20008198.2018.1472992 (PMC5965039; doi:10.1080/20008198.2018.1472992)
Supplement: Supplementary material [file ZEPT_A_1472992_SM0005.docx]

**Online supplement**

Table S1 *Correlations between PTSD and depressive symptoms*

|  | ***M*** | ***SD*** | **1** | **2** | **3** | **4** | **5** | **6** | **7** |
| --- | --- | --- | --- | --- | --- | --- | --- | --- | --- |
| **PTSD T1** | 23.35 | 11.34 | 1 |  |  |  |  |  |  |
| **PTSD T2** | 18.45 | 10.40 | .41^***^ | 1 |  |  |  |  |  |
| **PTSD T3** | 18.15 | 10.65 | .45^***^ | .68^***^ | 1 |  |  |  |  |
| **PTSD T4** | 18.12 | 10.74 | .41^***^ | .63^***^ | .64^***^ | 1 |  |  |  |
| **DEP T1** | 15.85 | 8.79 | .51^***^ | .36^***^ | .29^***^ | .38^***^ | 1 |  |  |
| **DEP T2** | 14.40 | 8.61 | .41^***^ | .70^***^ | .60^***^ | .53^***^ | .55^***^ | 1 |  |
| **DEP T3** | 14.63 | 9.29 | .44^***^ | .49^***^ | .67^***^ | .60^***^ | .44^***^ | .76^***^ | **1** |
| **DEP T4** | 14.44 | 9.08 | .34^***^ | .40^***^ | .46^***^ | .74^***^ | .41^***^ | .65^***^ | .78^*^**^**^** |

*Notes*. PTSD = Posttraumatic stress disorder; DEP = depressive symptoms; T1 = the first assessment 4 months after the earthquake; T2 = the second assessment 2.5 years after the earthquake; T3 = the third assessment 3.5 years after the earthquake; T4 = the fourth assessment 4.5 years after the earthquake.

^***^ *p* < .001

Table S2 *Loadings of the manifest indicators on their respective latent factors*

|  | DEP  T1 | PTSDT1 | DEP  T2 | PTSDT2 | DEP  T3 | PTSDT3 | DEP  T4 | PTSDT4 |
| --- | --- | --- | --- | --- | --- | --- | --- | --- |
| Anhedonia T1 | .76 |  |  |  |  |  |  |  |
| Negative Mood T1 | .80 |  |  |  |  |  |  |  |
| Negative Self-Esteem T1 | .77 |  |  |  |  |  |  |  |
| Ineffectiveness T1 | .63 |  |  |  |  |  |  |  |
| Interpersonal Problems T1 | .57 |  |  |  |  |  |  |  |
| Re-experiencing T1 |  | .80 |  |  |  |  |  |  |
| Avoidance T1 |  | .76 |  |  |  |  |  |  |
| Hyperarousal T1 |  | .76 |  |  |  |  |  |  |
| Anhedonia T2 |  |  | .79 |  |  |  |  |  |
| Negative Mood T2 |  |  | .83 |  |  |  |  |  |
| Negative Self-Esteem T2 |  |  | .76 |  |  |  |  |  |
| Ineffectiveness T2 |  |  | .67 |  |  |  |  |  |
| Interpersonal Problems T2 |  |  | .47 |  |  |  |  |  |
| Re-experiencing T2 |  |  |  | .74 |  |  |  |  |
| Avoidance T2 |  |  |  | .78 |  |  |  |  |
| Hyperarousal T2 |  |  |  | .82 |  |  |  |  |
| Anhedonia T3 |  |  |  |  | .87 |  |  |  |
| Negative Mood T3 |  |  |  |  | .86 |  |  |  |
| Negative Self-Esteem T3 |  |  |  |  | .78 |  |  |  |
| Ineffectiveness T3 |  |  |  |  | .73 |  |  |  |
| Interpersonal Problems T3 |  |  |  |  | .62 |  |  |  |
| Re-experiencing T3 |  |  |  |  |  | .83 |  |  |
| Avoidance T3 |  |  |  |  |  | .84 |  |  |
| Hyperarousal T3 |  |  |  |  |  | .83 |  |  |
| Anhedonia T4 |  |  |  |  |  |  | .91 |  |
| Negative Mood T4 |  |  |  |  |  |  | .55 |  |
| Negative Self-Esteem T4 |  |  |  |  |  |  | .76 |  |
| Ineffectiveness T4 |  |  |  |  |  |  | .68 |  |
| Interpersonal Problems T4 |  |  |  |  |  |  | .55 |  |
| Re-experiencing T4 |  |  |  |  |  |  |  | .77 |
| Avoidance T4 |  |  |  |  |  |  |  | .82 |
| Hyperarousal T4 |  |  |  |  |  |  |  | .86 |

*Notes*. PTSD = Posttraumatic stress disorder; DEP = depression; T1 = the first assessment 4 months after the earthquake; T2 = the second assessment 2.5 years after the earthquake; T3 = the third assessment 3.5 years after the earthquake; T4 = the fourth assessment 4.5 years after the earthquake.

All *p* < .001.

Table S3 Goodness-of-fit indices and model comparisons for tested models.

|  | S-Bχ^2^ | *df* | Δχ^2^ | Δ *df* | *p* | CFI | SRMR | BIC | RMSEA (90% CI) |
| --- | --- | --- | --- | --- | --- | --- | --- | --- | --- |
| UCLA PTSD-RI | | | | | | | | | |
| Configural invariance | 3364.963 | 2042 | — | — | — | .814 | .065 | 48125.243 | .046[.044-.049] |
| Metric invariance | 3428.239 | 2084 | 64.724 | 42 | .016 | .811 | .068 | 47948.821 | .046[.044-.049] |
| CDI | | | | | | | | | |
| Configural invariance | 8452.345 | 5318 | — | — | — | .670 | .066 | 51152.139 | .044[.042-.046] |
| Metric invariance | 8544.886 | 5384 | 92.541 | 66 | .017 | .668 | .068 | 50868.011 | .044[.042-.046] |


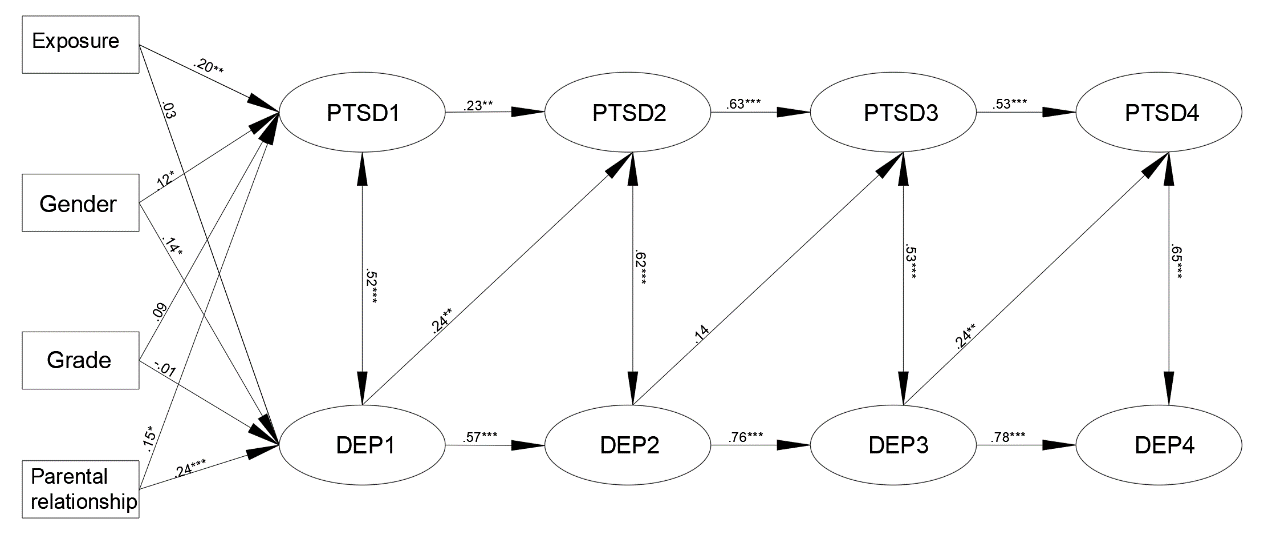


*Figure S1. The depressogenic model of PTSD and depression.*

*Notes*. PTSD = Posttraumatic stress disorder; DEP = depression; 1 = the first assessment 4 months after the earthquake; 2 = the second assessment 2.5 years after the earthquake; 3 = the third assessment 3.5 years after the earthquake; 4 = the fourth assessment 4.5 years after the earthquake.

^*^ *p* < .05

^**^ *p* < .01

^***^ *p* < .001


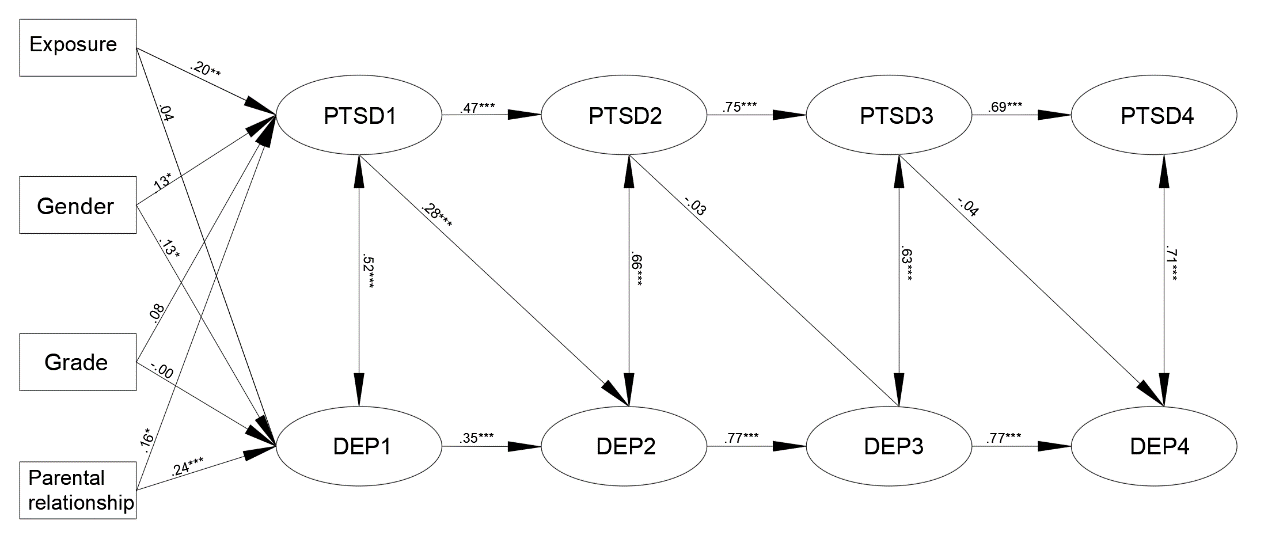


*Figure S2. The demoralization model of PTSD and depression.*

*Notes*. The same as above.
